# The role of muscle ultrasound in stroke rehabilitation: a review of calf muscle alterations and clinical implications

**DOI:** 10.3389/fneur.2026.1785196

**Published:** 2026-04-13

**Authors:** Ying Shi, Chuanxiong Li

**Affiliations:** Department of Rehabilitation Medicine, The Affiliated Hospital of Yunnan University, Kunming, Yunnan, China

**Keywords:** elastography, lower leg muscles, morphology, muscle spasticity, musculoskeletal ultrasonography, rehabilitation assessment, stroke

## Abstract

**Background:**

Post-stroke morphological and structural alterations in lower-leg muscles—including changes in muscle thickness, pennation angle, fascicle length, and echo intensity—are key factors contributing to gait impairment and functional disability in stroke survivors. Conventional clinical assessments, such as the Modified Ashworth Scale and Fugl-Meyer Assessment, are limited by subjectivity and an inability to quantify intramuscular structural changes. Musculoskeletal ultrasonography, as a non-invasive, real-time, and quantitative imaging tool, has emerged as a valuable technique for evaluating post-stroke muscle alterations.

**Objective:**

This systematic review aims to synthesize the literature published in the past 5 years on the application of musculoskeletal ultrasound—including B-mode, shear wave elastography, dynamic ultrasound, and quantitative ultrasound—in assessing morphological and functional changes of lower-leg muscles in stroke patients, and to explore its correlations with clinical outcomes and its utility in guiding rehabilitation interventions.

**Methods:**

PubMed, CNKI, and Wanfang Data were searched from January 2020 to December 2025 following the Preferred Reporting Items for Systematic Reviews and Meta-Analysis (PRISMA) guidelines. Studies were included if they were original research involving stroke patients, utilized ultrasound to assess lower-leg muscles, and reported at least one morphological or functional parameter.

**Results:**

A total of 8 studies published between 2020 and 2025 were included. The most frequently assessed muscles were the gastrocnemius and tibialis anterior. Compared with the non-paretic side or healthy controls, the paretic lower-leg muscles commonly exhibited reduced muscle thickness and cross-sectional area, altered pennation angle, shortened fascicle length, increased echo intensity (indicating fat infiltration or fibrosis), and elevated shear wave velocity or Young’s modulus (indicating increased stiffness). These ultrasound parameters showed significant correlations with clinical measures of spasticity (e.g., Modified Ashworth Scale), motor function (e.g., Fugl-Meyer Assessment), and muscle strength. Ultrasound was also effectively used to monitor treatment responses, including changes following botulinum toxin injection and rehabilitation training.

**Conclusion:**

Musculoskeletal ultrasonography is a promising imaging modality for objectively assessing structural and biomechanical alterations in lower-leg muscles after stroke. Recent evidence from the past 5 years confirms its value in providing insights into the pathophysiology of post-stroke muscle changes, correlating with functional outcomes, and guiding personalized rehabilitation. Future efforts should focus on establishing standardized imaging protocols to enhance clinical applicability and cross-study comparability.

## 1 Introduction

Stroke represents a major global public health challenge, with its high incidence, disability rate, and recurrence rate imposing a substantial burden on affected families and healthcare systems worldwide (1). In 2021, there were 11.946 million new stroke cases globally, with an age-standardized incidence rate (ASIR) of 142 per 100,000 population (2), with about 80% of survivors experiencing varying degrees of functional sequelae. Among these, lower limb dysfunction is prominent, affecting about 54% of patients and leading to significant gait abnormalities and an increased risk of falls (3). The lower-leg muscles—such as the gastrocnemius, soleus, and tibialis anterior—are crucial for gait and balance control. Post-stroke alterations in these muscles, including atrophy, fat infiltration, fibrosis, and spasticity, are key factors influencing functional recovery (4). Compared to conventional assessment tools like the Modified Ashworth Scale (MAS) and the Fugl-Meyer Assessment for lower extremities, which are limited by subjectivity and an inability to quantify intramuscular structural changes, ultrasonography offers an objective, quantifiable, convenient, and reproducible alternative, demonstrating considerable potential and clinical value in rehabilitation assessment (5).

## 2 Methods

### 2.1 Search strategy

Chinese search terms: (stroke OR cerebral apoplexy OR 卒中) AND (ultrasound OR elastography OR shear wave elastography OR musculoskeletal ultrasound) AND (lower leg muscles OR gastrocnemius OR tibialis anterior OR soleus). English search terms: (stroke OR cerebrovascular accident OR hemiplegia) AND (ultrasound OR ultrasonography OR elastography OR shear wave elastography OR muscle ultrasound) AND (lower leg OR calf OR gastrocnemius OR soleus OR tibialis anterior). We systematically searched PubMed, China National Knowledge Infrastructure (CNKI), and Wanfang Data for studies on the application of ultrasound in assessing lower leg muscle morphology and function in stroke patients. The search period was from January 2020 to December 2025.

### 2.2 Inclusion and exclusion criteria

Inclusion criteria:

1. Studies involving clinically diagnosed stroke patients;
2. Studies that utilized ultrasound imaging techniques (including B-mode ultrasound, shear wave elastography, strain elastography, dynamic ultrasound, or quantitative ultrasound) to assess lower leg muscles (gastrocnemius, soleus, tibialis anterior, etc.);
3. Studies that reported at least one muscle morphological or functional parameter (e.g., muscle thickness, cross-sectional area, pennation angle, fascicle length, echo intensity, shear wave velocity, Young’s modulus);
4. Original research articles (including cross-sectional studies, case–control studies, cohort studies, or interventional studies);
5. Full-text articles published in English or Chinese.

Exclusion criteria:

1. Reviews, conference abstracts, case reports, comments, or editorials;
2. Animal experiments or cadaveric studies;
3. Duplicate publications or studies with incomplete data;
4. Studies for which the full text could not be obtained.

### 2.3 Study selection and data extraction

Two reviewers independently screened the literature. Titles and abstracts were first reviewed to exclude obviously irrelevant studies, followed by full-text review of the remaining articles to determine final inclusion. Any disagreements were resolved through discussion or consultation with a third reviewer. The following information was extracted from the included studies: first author, publication year, study design, sample size, patient characteristics (age, time since stroke), muscles assessed, ultrasound technique, ultrasound parameters, main findings and Level of evidence.

### 2.4 Quality assessment

The quality of evidence for the included studies was assessed using the Oxford Centre for Evidence-Based Medicine (CEBM) 2009 levels of evidence. Based on study design (randomized controlled trial, cohort study, case–control study, case series, etc.) and study quality (randomization, blinding, loss to follow-up, control of confounding factors), each study was rated from level 1a to 5. Studies with significant methodological limitations were downgraded with a “-” suffix. The level of evidence for each study is indicated in Table 1.

**Table 1:** Methodological characteristics of studies.

No.	First author/year	Study design	Patient characteristics (age/time post-stroke)	Sample size(stroke patients)	Muscles assessed	Ultrasound technique	Ultrasound parameters	Main findings	Level of evidence
1	Liu et al. (35)	RCT	17-75 years/≤90 days	37	GL	B-mode ultrasound	MT PA	Monitoring of rehabilitation efficacy	1b
2	Chen et al. (16)	RCT	25-80 years/1 month-2 years	64	GL、TA	SWE	SWV YM	Monitoring of rehabilitation efficacy	1b
3	Mori et al. (27)	Cohort study	40-70 years/≤40 weeks	38	TA	B-mode ultrasound	EI	Clinical assessment of spasticity severity	2b
4	Dang et al. (6)	RCT	30-80 years/<6 months	180	TA	B-mode ultrasound	MT PA FL	Monitoring of rehabilitation efficacy	1b
5	Cao al. (26)	Cohort study	40-70 years/<6 months	19	GL	SWE	SWV YM	Clinical assessment of spasticity severity	2b
6	Zhu et al. (19)	RCT	30-70 years/<12 months	60	GL	B-mode ultrasound	CSA MT PA	Monitoring of rehabilitation efficacy	1b
7	Shu et al. (30)	Cohort Study	33-65 years/<1-11 months	44	SOL、GL	B-mode ultrasound SWV	MGV CSA MT PA SWV	Clinical assessment of spasticity severity	2b
8	Chen et al. (32)	Cohort Study	40-70 years/<6 months	98	GL	SWE	SWV YM	Clinical assessment of spasticity severity	2b

GL, gastrocnemius lateralis; TA, tibialis anterior; SOL, Soleus; PA, pennation angle; MGV, Medial gastrocnemius volume; CSA, Cross-sectional area; MT, Muscle thickness; YM, Young’s modulus; EI, Echo intensity; SWE, Shear wave elastography; SWV, Shear wave velocity; RCT, Randomized Controlled Trial.

### 2.5 Limited number of included studies

This systematic review identified only eight studies that met the inclusion criteria. This relatively small number reflects the stringent selection criteria applied, including the focus on lower leg muscles, the requirement for ultrasound-based assessment, and the restriction to studies published between 2020 and 2025. While this limited evidence base may affect the generalizability of our conclusions, it also underscores the novelty of this research area and highlights the need for further high-quality investigations. Future studies with larger sample sizes and standardized protocols are warranted to strengthen the evidence.

## 3 Overview of pathophysiological changes in lower leg muscles after stroke

### 3.1 Neurogenic alterations

Upper motor neuron injury following stroke disrupts the excitatory signaling from the cerebral cortex to the anterior horn motor neurons in the spinal cord, leading to a loss of voluntary and active contraction capacity in the innervated muscles—a state often referred to as central paresis or loss of supraspinal drive.” (6). In the early phase of loss of supraspinal drive, the removal of central inhibitory inputs may result in abnormally increased intrinsic excitability of the spinal motor neurons. This is clinically manifested as hypertonia and hyperreflexia, commonly referred to as spasticity. It should be noted, however, that such spasticity represents a passive and inefficient neural activity, which does not translate into functional movement.

### 3.2 Changes in muscle structure and mechanical properties

#### 3.2.1 Morphological changes

**Figure 1:**
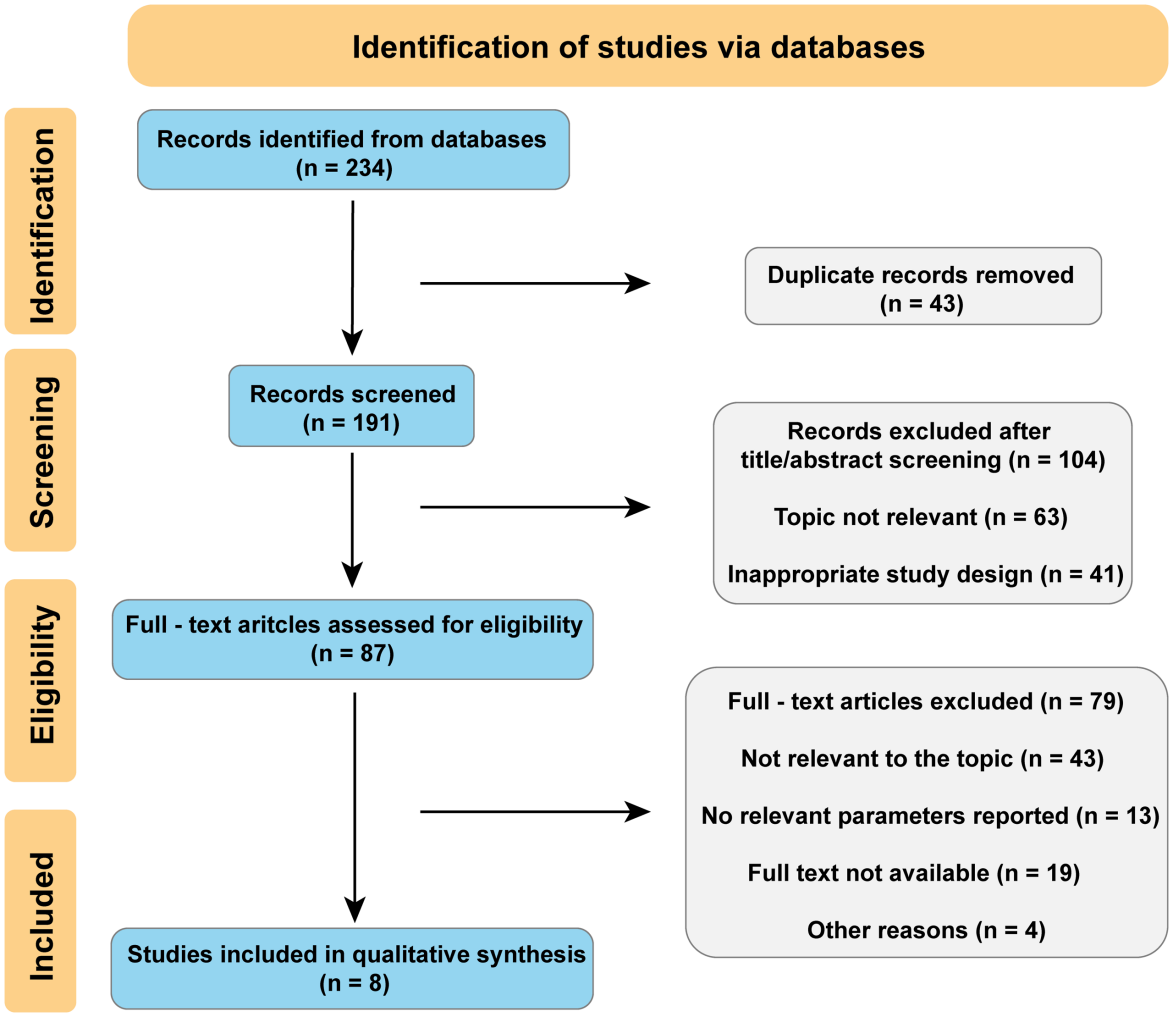
PRISMA 2020 flow diagram.

Ultrasonography enables the extraction of multiple morphological parameters that reflect the functional status of muscles, primarily including muscle thickness, pennation angle, and muscle fiber length (7). The pennation angle refers to the angle between muscle fibers and the tendon; a larger angle allows for more muscle fibers within a given muscle volume, thereby contributing to greater force generation (8). In the paretic lower leg after stroke, the gastrocnemius muscle, which is often predominantly spastic, frequently exhibits an increased pennation angle. Conversely, in severely atrophied or predominantly weak muscle groups, the pennation angle may decrease. Muscle thickness (often represented as cross-sectional area) serves as a fundamental indicator for assessing muscle mass. Its standardized measurement can quantify the loss of muscle volume, although it does not directly reflect contractile function (9). Studies have shown that the thickness and cross-sectional area of both the gastrocnemius and tibialis anterior muscles are significantly smaller on the paretic side compared to the non-paretic side in stroke patients. Muscle fiber length, defined as the total length of fibers extending between the deep and superficial fasciae, provides insight into the intrinsic architecture of the muscle and indirectly suggests its mechanical properties. Alterations in muscle fiber morphology represent a potential mechanism affecting force transmission through the tendon and are closely related to myocellular structure and musculo-skeletal alignment (10). In the paretic lower leg after stroke, fascicle length is often shortened (Figure 1).

#### 3.2.2 Textural changes

Post-stroke alterations also occur in the texture of lower leg muscles. Increased echo intensity, a key feature in musculoskeletal ultrasonography, primarily indicates fat infiltration or fibrosis within the muscle tissue (11). Higher echo intensity typically suggests a more severe degree of muscle fatty degeneration or fibrosis, signifying a decline in muscle quality.

#### 3.2.3 Biomechanical changes

The aforementioned morphological and textural alterations collectively lead to a deterioration in the macroscopic biomechanical properties of the muscle, manifesting as increased stiffness, reduced compliance, and clinically prominent spasticity. Following a stroke, the paretic muscles exhibit reduced volume and a decreased number of functioning motor units. These structural changes are highly correlated with muscle weakness, spasticity, and the development of contractures (4).

## 4 Application of ultrasonographic assessment techniques in post-stroke lower leg muscle research

### 4.1 Musculoskeletal ultrasonography

Musculoskeletal ultrasonography is an imaging technique based on ultrasound principles that is used to assess the mechanical properties of tissues. By employing high-frequency transducers to detect alterations in muscles and surrounding soft tissues, it provides quantitative information for an objective description of muscular physical properties (12). Currently, the main ultrasonographic techniques used in clinical practice include B-mode ultrasound, shear wave elastography, dynamic/real-time ultrasound, and quantitative ultrasound.

### 4.2 B-mode ultrasound

B-mode ultrasound is the most fundamental and widely used imaging tool for assessing the static morphological structure of muscles. It enables the precise quantification of a series of morphological changes in lower leg muscles following stroke, including muscle thickness, pennation angle, fascicle length, and echo intensity, thereby providing an objective basis for clinical evaluation.

#### 4.2.1 Muscle thickness

Muscle thickness is a core indicator for quantifying muscle atrophy. Measurements taken with B-mode ultrasound at standardized anatomical sites (e.g., the maximal girth of the gastrocnemius muscle belly) can clearly demonstrate a significant reduction in muscle thickness and cross-sectional area on the paretic side compared to the non-paretic side in stroke patients (13).

#### 4.2.2 Pennation angle

The pennation angle serves as a structural basis for evaluating muscular force-generation efficiency. A reduction in this angle is associated with declines in muscle mass and function. On the paretic side after stroke, due to muscle fiber shortening and increased resting tension, the gastrocnemius muscle—often predominantly spastic—may exhibit characteristic changes in its pennation angle, consequently affecting **its** force-production efficiency (14).

#### 4.2.3 Fascicle length

Fascicle length reflects the maximum contraction amplitude and speed of a muscle. Prolonged immobilization and spasticity after stroke predispose muscles to adaptive shortening and fibrosis, leading to a significant reduction in fascicle length on the paretic side (13).

#### 4.2.4 Echo intensity

Changes in echo intensity indicate alterations in muscle texture. Following a stroke, atrophied muscle fibers are often replaced by adipose and fibrous connective tissues. These tissues interact more strongly with ultrasound waves, generating more echo signals, which manifests as increased echo intensity (15).

### 4.3 Shear wave elastography

Shear wave elastography quantifies tissue stiffness by measuring the propagation speed of shear waves within it. The elastic modulus or stiffness of biological tissue is closely related to its molecular composition and microstructure, with differences often existing between pathological and normal tissues; shear waves propagate faster in stiffer tissues. Key quantitative parameters of this technique include shear wave velocity and Young’s modulus. Shear wave velocity directly reflects tissue stiffness, with higher values indicating greater muscle stiffness (15). Young’s modulus (typically expressed in kilopascals) is proportional to tissue stiffness and provides another quantitative measure of hardness. Studies have found that the SWV of lower leg muscles (e.g., the gastrocnemius) on the paretic side is often significantly higher than that on the non-paretic side in stroke patients (16).

### 4.4 Dynamic/real-time ultrasound

Dynamic ultrasound allows for the real-time recording of structural changes in muscles while patients perform specific functional tasks, such as walking or active contraction (17). Its primary advantage lies in directly linking morphological assessment with real-time functional performance, thereby revealing muscle working mechanisms and coordination patterns that cannot be captured by static imaging (17).

### 4.5 Quantitative ultrasound

Quantitative ultrasound employs computer-assisted mathematical algorithms to perform quantitative analysis of textural features in ultrasound images. It can detect subtle structural changes indistinguishable to the human eye (such as early-stage fat infiltration and fibrosis), providing a more sensitive and objective tool for assessing microstructural alterations in lower leg muscles after stroke (18).

## 5 Correlation between ultrasonographic parameters and clinical function

### 5.1 Correlation with spasticity severity

Multiple studies indicate quantitative associations between ultrasonographic morphological/elastic parameters and the severity of post-stroke muscle spasticity. Zhu et al. (19) found that changes in muscle strength following the onset of spasticity in stroke patients could lead to a reduction in the pennation angle, suggesting this parameter may be useful for assessing spasticity severity. Swanson et al. (20) proposed that the combined measurement of muscle cross-sectional area and thickness can serve as an effective clinical indicator for limb spasticity. Regarding elastography, Young’s modulus is a key parameter for quantifying muscle spasticity. Increased tissue stiffness and density result in faster shear wave propagation and correspondingly higher Young’s modulus values. Studies confirm that the shear wave velocity in spastic muscles of stroke patients is significantly higher than that in the non-paretic side and in healthy individuals (21). Research by Vigotsky et al. (22) further demonstrated a positive correlation between shear wave velocity and joint stiffness in spastic limbs. Conventional subjective clinical assessments are limited in their ability to directly quantify spasticity, as they cannot differentiate whether its predominant component arises from neural hyperexcitability or structural remodeling. Neural hyperexcitability refers to the velocity-dependent increase in muscle tone resulting from exaggerated stretch reflexes, typically assessed using clinical scales such as the Tardieu Scale or the Modified Ashworth Scale. In contrast, structural remodeling encompasses passive biomechanical changes within the muscle itself, including fibrosis, fat infiltration, and increased stiffness, which can be quantitatively evaluated using shear wave elastography. While clinical scales reflect the combined effects of neural and biomechanical components, SWE provides a direct quantitative measurement of the passive mechanical properties of muscle. Therefore, SWE is particularly valuable in the following scenarios: when it is necessary to determine whether increased resistance to passive movement is primarily neurogenic or myogenic in origin; when clinicians need to monitor structural changes over time (e.g., following botulinum toxin injections or rehabilitation training); or when researchers aim to quantify the effects of interventions on muscle tissue itself, independent of reflex-mediated contributions. The combined use of SWE and clinical scales enables a comprehensive assessment of spasticity, thereby informing the development of more targeted and individualized treatment strategies.

### 5.2 Correlation with motor function and walking ability

Morphological alterations in lower leg muscles are closely related to scores on lower limb motor function assessments. Studies show that muscle thickness and cross-sectional area of the tibialis anterior are significantly positively correlated with the Fugl-Meyer Assessment score for the lower extremities (23). The Timed Up and Go test (TUG) is a comprehensive measure of balance, mobility, and functional walking. Research indicates that the muscle thickness of the medial gastrocnemius is negatively correlated with TUG completion time. Additionally, excessively high muscle stiffness can limit joint mobility and impair coordination, leading to prolonged TUG times (24). The 10-Meter Walk Test (10MWT) is the gold standard for assessing walking speed, a key parameter of functional mobility. It has been suggested that post-stroke changes in the pennation angle may reflect reduced muscle force-generation efficiency, directly impacting propulsion and final walking speed (25). Shear wave elastography, as a quantitative tool, holds significant value in assessing spastic hypertonia. Cao et al. (26), published in the Journal of NeuroEngineering and Rehabilitation, demonstrated that the 2D-SWE-derived parameter (corrected slack angle) of the medial gastrocnemius muscle in stroke patients showed a significant negative correlation with MAS scores (R = −0.849, *p* < 0.001), confirming the reliability of SWE in quantifying post-stroke spasticity.

### 5.3 Correlation with muscle strength

Muscle cross-sectional area and thickness are fundamental determinants of muscle strength. Research demonstrates a significant positive correlation between the cross-sectional area of the tibialis anterior and isometric dorsiflexion strength (27). The pennation angle, a core structural parameter for evaluating force-generation efficiency, shows a positive correlation with plantar flexion strength of the soleus in healthy individuals (28). Following stroke, however, muscle disuse and weakness are often accompanied by a decreased pennation angle. Echo intensity, an indicator of muscle fat infiltration and fibrosis, is negatively correlated with isometric muscle strength (29). Higher echo intensity suggests poorer muscle quality and correspondingly lower force output.

## 6 Application of ultrasonography in assessing rehabilitation outcomes

### 6.1 Evaluating the efficacy of botulinum toxin therapy

Ultrasonography provides a quantitative means to assess muscular alterations in stroke patients with lower limb spastic paralysis, serving as an objective and effective tool for evaluating the therapeutic outcomes of botulinum toxin (BoNT) injection, thereby holding significant clinical value (30). Research indicates that ultrasonographic parameters are sensitive to changes in both muscle morphology and elasticity following treatment. For instance, a study on post-stroke upper limb spasticity revealed that the mean Young’s modulus of target muscles, such as the flexor digitorum superficialis and profundus, showed a continuous decrease at 1 and 3 months after BoNT-A injection, a trend consistent with improvements in the Modified Ashworth Scale scores (31). Another study focusing on lower limb spasticity confirmed that BoNT-A injection guided by shear wave elastography, combined with systematic rehabilitation training, can more effectively alleviate muscle spasticity and improve patients’ balance and motor function (32). It is noteworthy that long-term BoNT-A therapy may lead to disuse muscle atrophy. Regular monitoring of changes in muscle thickness and cross-sectional area using B-mode ultrasound enables clinicians to precisely quantify the progression of atrophy. This information is crucial for adjusting injection dosages and intervals and formulating individualized treatment plans (32).

### 6.2 Dynamic monitoring of rehabilitation efficacy

Studies have confirmed that effective rehabilitation training can induce positive adaptive changes in muscle architecture. Following a stroke, the pennation angle of the paretic gastrocnemius and tibialis anterior muscles is often significantly smaller than that on the non-paretic side. Post-rehabilitation increases in pennation angle accompanied by gains in muscle thickness suggest morphological hypertrophy and remodeling (33). For example, Liu et al. (34) demonstrated that after 3 weeks of body-weight-supported treadmill training, stroke patients showed an average increase of 1.2° in the pennation angle of the tibialis anterior, which was significantly correlated with improved muscle strength (*p* < 0.05). Other research indicates that electromyographic biofeedback therapy can enhance lower limb balance ability by improving the morphological structure (e.g., pennation angle and thickness) of the gastrocnemius muscle (35). Various rehabilitation modalities have shown positive effects on muscle structure. Ghasemi et al. (36) implemented a 4-week functional stretching exercise program for chronic stroke patients and confirmed its beneficial impact on the architecture of the gastrocnemius muscle. Shao et al. (37) suggested that combining conventional ultrasound with elastography to observe changes in Achilles tendon length, thickness, and elasticity can effectively assess tendon condition post-stroke and monitor recovery following rehabilitation training. Regarding modern rehabilitation technologies, a study by Song et al. (38) found that after 4 weeks of rehabilitation robot training, patients in the subacute phase of stroke exhibited significant increases in pennation angle, thickness, and fascicle length of the paretic tibialis anterior and medial gastrocnemius muscles, along with concurrent improvements in walking function.

## 7 Current limitations and future perspectives

### 7.1 Technical limitations

Despite its considerable potential in assessing post-stroke muscular alterations, the application of musculoskeletal ultrasonography faces several technical challenges.

#### 7.1.1 Operator dependence

The reliability of ultrasonographic measurements, particularly for sensitive parameters like the pennation angle, is highly dependent on the operator’s skill and experience. Studies show significant differences in inter-rater agreement between experienced and novice examiners. Subtle variations in probe placement angle, position, and applied pressure can introduce substantial random errors, compromising the reproducibility and comparability of results (39).

#### 7.1.2 Consistency across devices and measurements

Quantitative values derived from ultrasonography, especially shear wave velocity and Young’s modulus from elastography, are significantly influenced by the equipment manufacturer, transducer model, and software algorithms. This variability hinders direct comparison of data acquired from different systems. Furthermore, as anisotropic tissues, muscle elastic moduli change with probe angle and limb position, adding to the difficulty of standardizing data across research centers (40).

#### 7.1.3 Lack of standardization in image analysis

Currently, there is a lack of globally unified standards for key aspects of muscle ultrasound, including image acquisition protocols, patient positioning, transducer selection, parameter settings, and post-processing analysis methods. Additionally, inherent anatomical variations in lower leg muscles (e.g., accessory soleus) may be misinterpreted as pathological findings during examination, necessitating solid anatomical knowledge from the operator to avoid misdiagnosis (41).

#### 7.1.4 Challenges in imaging deep muscles

While ultrasound provides excellent imaging of superficial muscles, assessing deeper muscles such as the tibialis posterior and flexor digitorum longus remains challenging. Signal attenuation in deeper tissues reduces image resolution and clarity, potentially affecting the accuracy of morphological and elastic parameter measurements (41).

### 7.2 Research limitations

Beyond technical factors, existing studies in this field also have inherent limitations.

#### 7.2.1 Generally small sample sizes

Many exploratory studies are limited by small sample sizes, resulting in low statistical power. Their findings are susceptible to influence from outliers and may not accurately reflect the true effect size, limiting the generalizability of conclusions.

#### 7.2.2 Scarcity of long-term longitudinal data

Current research primarily focuses on muscular changes during the acute or subacute phases post-stroke. There is a notable lack of longitudinal data on the long-term evolution of muscle structure in the chronic phase. Critical questions regarding the fate of lower leg muscles after hospital discharge—such as whether atrophy progresses, fat infiltration continues, or if community-based rehabilitation can reverse these changes—remain unanswered due to the absence of long-term follow-up studies.

#### 7.2.3 Incomplete understanding of the link between muscle structure and neural control

Although studies have established correlations between certain ultrasonographic parameters and clinical functional scores, these associations largely remain statistical. The underlying physiological and biomechanical links between alterations in muscle morphology/mechanical properties and changes in motor control strategies (e.g., motor unit recruitment patterns, muscle co-activation) following central nervous system injury are not yet fully understood. For instance, the extent to which reduced muscle stiffness directly contributes to improved motor function requires clarification through more sophisticated multimodal studies integrating ultrasonography, electromyography, and motion analysis.

### 7.3 Future perspectives

To address current limitations and advance the field, future efforts should focus on the following directions.

#### 7.3.1 Promotion of multimodal technology integration

Integrating B-mode ultrasound (high-resolution morphology), ultrasound elastography (quantitative stiffness assessment), and dynamic/real-time ultrasound (real-time functional observation) can establish a comprehensive “structure-mechanics-function” assessment framework (17). For example, simultaneous acquisition of morphological and elastographic data during gait analysis can reveal the complete dynamics of muscle changes throughout the gait cycle, providing a richer evidence base for developing highly individualized rehabilitation plans.

#### 7.3.2 Establishment of standardized protocols and consensus

A major bottleneck in the field’s development is the absence of unified standards. There is an urgent need to develop international or professional consensus guidelines for the ultrasonographic assessment of lower leg muscles in stroke patients. It including: Standardized anatomical landmarks for probe placement; Patient positioning and joint angle during measurement; Ultrasound device and transducer specifications; Measurement protocols for each parameter (e.g., thickness, pennation angle, SWV); Reporting of intra- and inter-rater reliability.

## 8 Conclusion

Owing to its unique advantages of being non-invasive, real-time, quantitative, and capable of multimodal integration, ultrasonography has become a pivotal bridge connecting the investigation of post-stroke muscle pathophysiological mechanisms with clinical rehabilitation practice. With continued technological advancements and the refinement of application standards, it is poised to evolve into a routine assessment tool in stroke rehabilitation. This will provide indispensable objective evidence for enhancing the precision and efficacy of rehabilitation interventions, ultimately improving functional outcomes and the quality of life for patients.

## Data Availability

The original contributions presented in the study are included in the article/supplementary material, further inquiries can be directed to the corresponding author.
